# Supplementary material for: Regulators of TNFα mediated insulin resistance elucidated by quantitative proteomics
Source: Sci Rep. 2020 Nov 30;10:20878. doi: 10.1038/s41598-020-77914-1 (PMC7705713; doi:10.1038/s41598-020-77914-1)
Supplement: Supplementary file 1 — Supplementary Figures. [file 41598_2020_77914_MOESM1_ESM.pdf]

Regulators of TNFα Mediated Insulin Resistance Elucidated by Quantitative Proteomics

Rodrigo Mohallem[1,2] and Uma K. Aryal[1,2*]
[1]Department of Comparative Pathobiology, Purdue University, West Lafayette, United States. [2]Purdue Proteomics Facility, Bindley Bioscience Center, Purdue University, West Lafayette, United States.
*Corresponding Author: uaryal@purdue.edu

Figure S1. Experimental Design and Label-Free Quantitative Secretomic and Phosophoproteomic Analysis Workflow. (A) Spend media was collected and concentrated. Protein was extracted, with sequential reduction and alkylation of cysteine disulfide bonds. Proteins were digested with Lys-C/Trypsin, desalted and analyzed by HPLC-MS/MS. (B) Protein was extracted, with sequential reduction and alkylation of cysteine disulfide bonds. Proteins were digested with Lys-C/Trypsin, desalted, and phosphopeptides were enriched before HPLC-MS/MS analysis.

Figure S2. Treatment groups are highly correlated: (A) PCA plot depiction of all replicates analyzed in the global proteomics. Individual samples are indicated by color, indicated in the figure legend. (B) Scree plot representation of Principal Components.

Figure S3. Secretome analysis of TNFα chronically treated adipocytes. (A) Heat map of all quantified proteins for both 4D and 8D time points. Color scale represents Z-scored LFQ values. (B) Volcano plots of all quantified proteins in each time point. Horizontal red line represents the Log_{10} (p value) cutoff, and vertical blue lines represent the Log_2 (Fold Change) cutoff.

Figure S4. Full-length blots of Csf1, Actin Mif expression levels in 4D and 8D TNFα treated adipocytes 4.

Figure S1

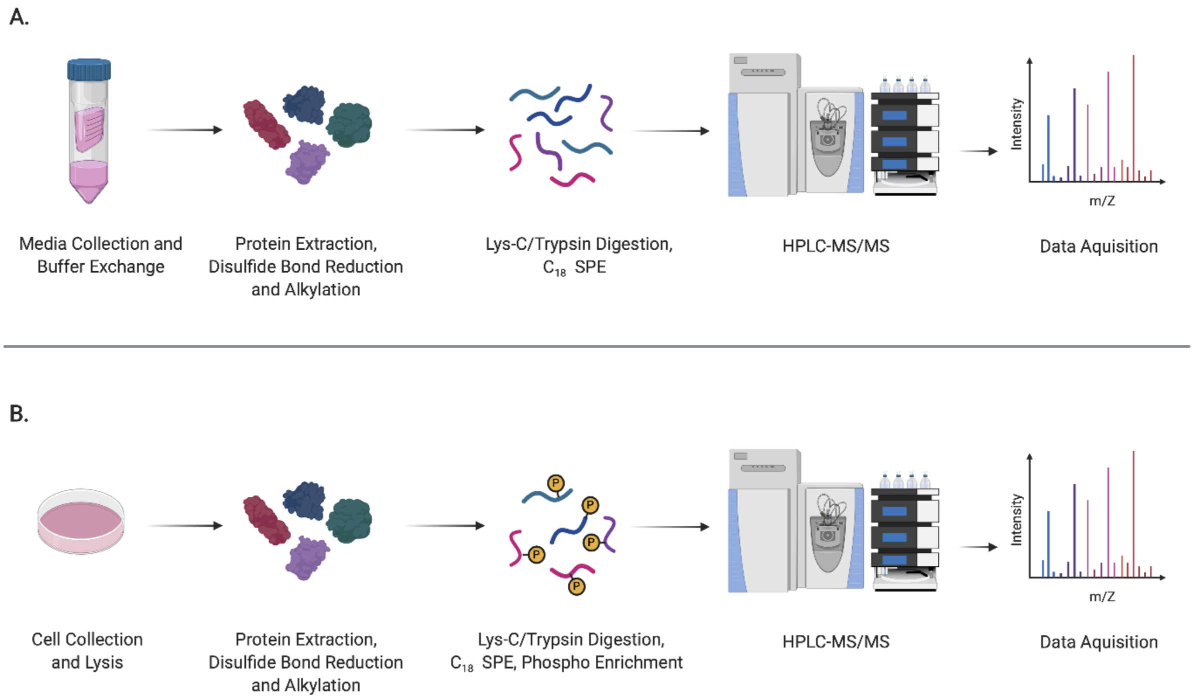
A.
Intensity
m/Z
Media Collection and Buffer Exchange
Protein Extraction, Disulfide Bond Reduction and Alkylation
Lys-C/Trypsin Digestion, C_{18} SPE
HPLC-MS/MS
Data Aquisition
B.
P
Intensity
m/Z
Cell Collection and Lysis
Protein Extraction, Disulfide Bond Reduction and Alkylation
Lys-C/Trypsin Digestion, C_{18} SPE, Phospho Enrichment
HPLC-MS/MS
Data Aquisition

Figure S2

A.

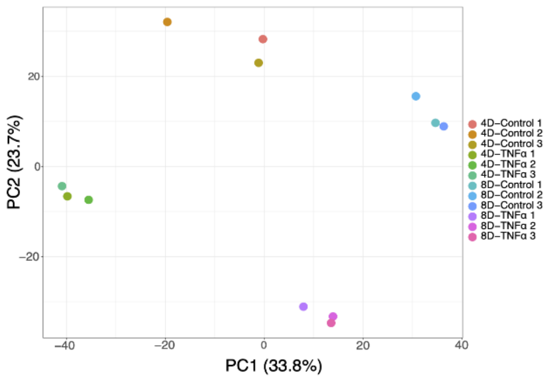
PC2 (23.7%)
PC1 (33.8%)
20
0
−20
−40
−20
0
20
40
4D−Control 1
4D−Control 2
4D−Control 3
4D−TNFα 1
4D−TNFα 2
4D−TNFα 3
8D−Control 1
8D−Control 2
8D−Control 3
8D−TNFα 1
8D−TNFα 2
8D−TNFα 3

B.

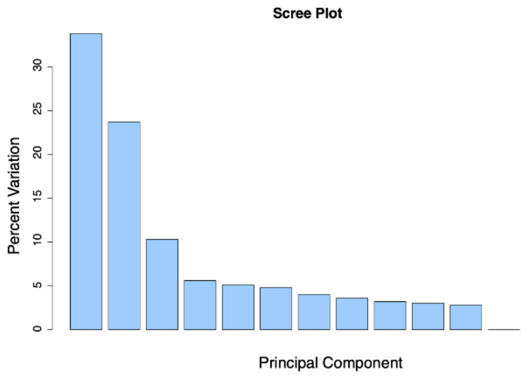
Scree Plot
Percent Variation
0
5
10
15
20
25
30
Principal Component

Figure S3

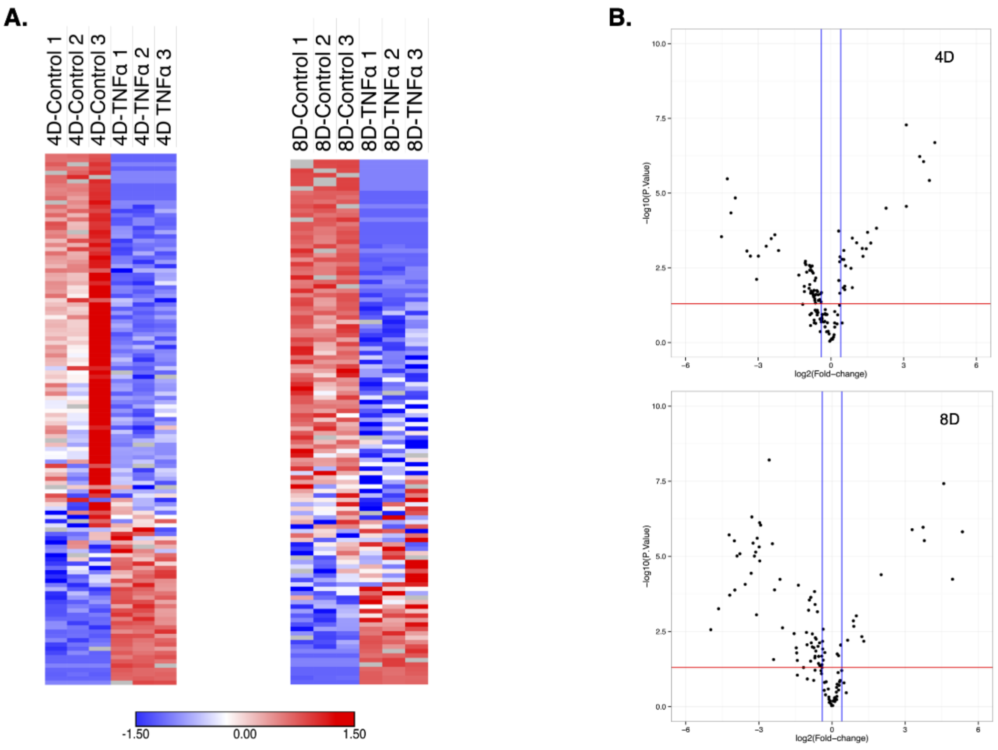
A.
4D-Control 1
4D-Control 2
4D-Control 3
4D-TNFα 1
4D-TNFα 2
4D TNFα 3
8D-Control 1
8D-Control 2
8D-Control 3
8D-TNFα 1
8D-TNFα 2
8D-TNFα 3
-1.50
0.00
1.50
B.
4D
8D
−log10(P.Value)
log2(Fold−change)

Figure S4

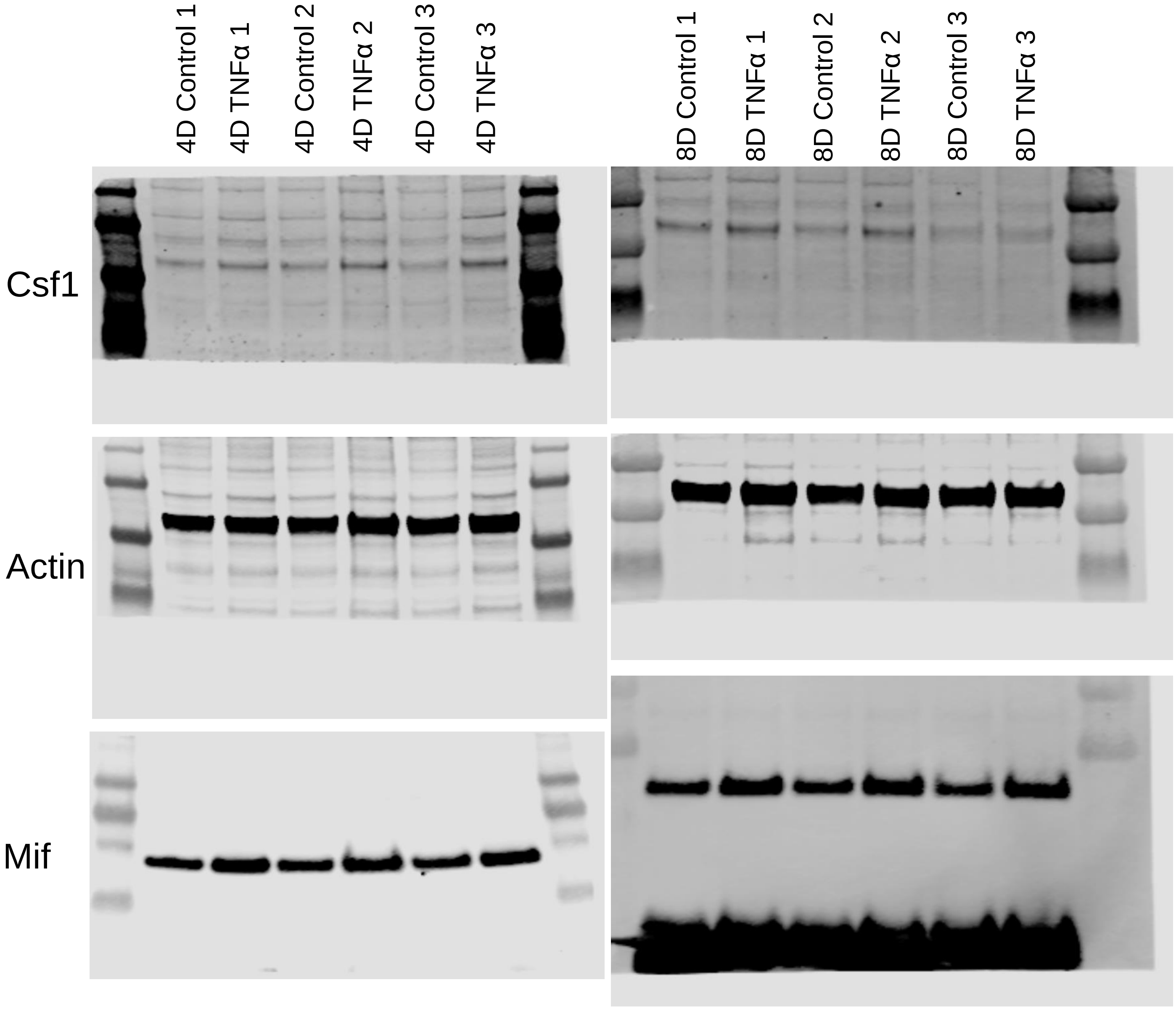
4D Control 1
4D TNFα 1
4D Control 2
4D TNFα 2
4D Control 3
4D TNFα 3
8D Control 1
8D TNFα 1
8D Control 2
8D TNFα 2
8D Control 3
8D TNFα 3
Csf1
Actin
Mif
